# Evidence That Jeanne Calment Died in 1934—Not 1997

**DOI:** 10.1089/rej.2018.2167

**Published:** 2019-02-28

**Authors:** Nikolay Zak

**Affiliations:** Moscow Society of Naturalists, Moscow, Russia.

**Keywords:** Jeanne Calment, mortality plateau, supercentenarians, validation

## Abstract

I present a body of data that, I argue, cumulatively casts serious doubt on the validity of Jeanne Calment's accepted world record of human life span. First, I assess the plausibility of the record based on the life spans of other centenarians in the International Database of Longevity (IDL) and critique some arguments put forward previously in support of that plausibility, including the longevity of Calment's ancestors. Second, I review the literature dedicated to Calment and discuss multiple contradictions in her interviews, biographies, photos, and documents. I argue that the evidence from these sources motivates renewed consideration of the previously rejected hypothesis that Jeanne's daughter Yvonne acquired her mother's identity after her death to avoid financial problems and that Jeanne Calment's death was reported as Yvonne's death in 1934. Finally I discuss the importance of reconsidering the principles of validation, due to the possibility of similar problems regarding other exceptionally long-lived people and the mistaken inferences that researchers may draw from flawed datasets. The phenomenon of Jeanne Calment may prove to be an instructive example of the uncertainty of seemingly well-established facts.

## Evaluation of the Plausibility of Jeanne Calment's Life Span

### Demographic considerations

Much attention has recently been devoted to the dynamics of mortality in older ages (105 years and older).^1–6^ A detailed analysis of these articles, and explanations for the causes of the disagreements between some of them, was provided in Zak, 2018.^7^ Some researchers conclude that, for whatever reason, the force of mortality progressively departs from the celebrated Gompertz formula at extreme ages and is almost constant after 105 years (and also nearly invariant with sex, country, and year of birth), while others conclude that the force of mortality continues to rise with age. None of these studies, however, has identified any *decline* in the force of mortality with age, at least until the data become too scarce to make a determination. (This is in intriguing contrast to what has been observed in insects.) However, Calment's age at death exceeds that of the current runner-up (Sarah Knauss) by more than 3 years; thus, at a first glance she looks like an exception.^8^ But how extreme an exception?

We can quantify the likelihood that the last survivor of a group reaches a given age using Monte Carlo simulation. We make the conservative assumption that the force of mortality does indeed stay constant from age 110, and we use a value for that annual mortality rate of 0.5, which is what is observed in (*e.g.*) the Italians born in 1904 and alive in 2009, approximately half of whom died during any year of follow-up observation through to 2016.^4^ Under this assumption, the probability of the event that anybody from a group consisting of *N* supercentenarians would be alive after *t* years is:

\begin{align*}
P \left( {t , N} \right) \; = \;1 \; - \;{ \left( {1 \; - \;{{0.5}^t}} \right) ^N}.
\end{align*}

Jeanne Calment was born on February 21, 1875 and had been alive for 12 years and 164 days (12.45 years) after her 110th anniversary. All the other 48 supercentenarians in the French section of the International Database of Longevity (IDL) containing verified supercentenarians^9^ listed on www.supercentenarians.org were born later than the person under the ID number 584, whose life span corresponds to that of Jeanne Calment.

Thus, the first (by date of birth) French woman to be validated as a supercentenarian by a large project searching for long-lived people (funded by the Ipsen foundation^10^) achieved the world record. Since IDL is free from age ascertainment bias,^9^ one can estimate the plausibility of the emergence of the record in IDL by 1998 by considering the group consisting of all 80 individuals in the IDL dataset born before 1876. Then

\begin{align*}
P \left( {t , N} \right) \; = \;1 \; - \;{ \left( {1 \; - \;{{0.5}^{12.45}}} \right) ^{80}} \; \approx \;0.014.
\end{align*}

**FIG. 1.**
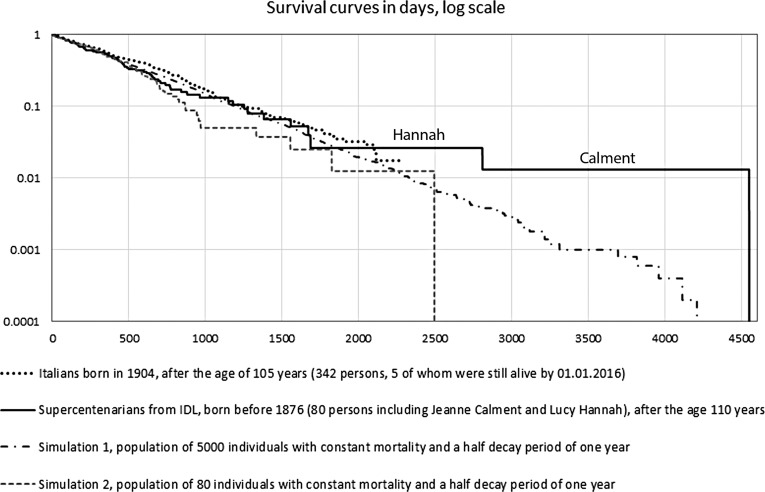
Survival curves of the cohort of Italians (ISTAT) after 105 years, the group of supercentenarians from IDL born before 1876 after 110 years (Jeanne Calment died last), and representative simulated populations with a constant mortality rate. IDL, International Database of Longevity.

I performed a Monte Carlo simulation of this equation. The longest-lived member of the simulated population consisting of 80 individuals who have a constant force of mortality, and half of whom die every year, did not live as long as 12.45 years. Indeed, this was the case even when the initial population size was increased to 5000, which is greater than the number of people validated to have reached 110 worldwide even as of today. By contrast, the survival curve of the Italian cohort after 105 years (ISTAT data) and the survival curve of the IDL group above without Calment do not differ much from the simulations (Fig. 1).

Incidentally, the second place in this group (and the first one among all the validated long-lived people of African descent) belongs to Lucy Hannah (validated age at death 117 years and 248 days). Similar to the majority of the U.S. supercentenarians in the IDL,^2^ her validation was based on archive data, such as censuses, which may not be reliable age confirmation.^11^ Independent researchers from the 110 Club forum discovered that she was probably 20 years younger than her claim,^12^ but she is still present in the IDL and Gerontology Research Group (GRG) databases.

### Genetic considerations

Myriam Provence studied lifespans of Jeanne's ancestors and noticed the positive trend in time. She hypothesized that her longevity could be inherited.^49^ Similarly, the researchers who conducted the validation of Calment's age, Jean-Marie Robine and Michel Allard, attribute her exceptional life span to her extraordinary genes inherited from her presumably exceptional ancestors.^13^ They write: “Many ancestors of Madame Calment could have been unconsciously chosen (by the spouse or by their families) because they were carriers of genes favoring longevity. Gradually, there would have been a concentration, a unique enrichment of this type of gene, to permit such a long existence.”^14^ In an article^13^ devoted to justifying the life span of Jeanne Calment by reference to the longevity of her ancestors, Robine and Allard state that “One might hypothesize that the extraordinary longevity of Jeanne Calment is largely genetic in origin and that it is due to an exceptional genetic inheritance, randomly accumulated within the ecological niche of Arles in the 18th and 19th centuries (and, more specifically, within the social group of craftsmen and *shopkeepers* [sic, see section “Who owned a store?”] running prosperous businesses in the town).” Similarly: “Examination of her paternal and maternal ascendants [sic] show at least three generations of exceptional longevity.”^15^

Ostensibly supporting this conclusion, the validators studied the longevity of many generations of Jeanne Calment's relatives, comparing them to the control group of residents of Arles, and found that “infant mortality was 27% in controls compared to 15% in the Calment group; the life expectancy of the residents of Arles was 27 years against the 41 years in the Calment group; the direct ancestors of Jeanne lived on average 80 years compared to only 58 years for the ancestors of other members of her family of the same generation.”^14,16^

Unfortunately, these authors do not provide the raw life span data of Calment's ancestors. Thus, with the help of French researchers Arnaud and Cyril^62^ from a forum on French centenarians,^17^ I obtained the life span data for four generations of the ancestors of Jeanne, consisting of 30 persons born between 1723 (Vincent Calment, great-grandfather of Nicolas Calment) and 1838 (Marguerite Gilles, wife of Nicolas Calment). The average life span of these ancestors appears to be 72.3 years, somewhat less than the declared 80 but still higher than 58.

I thus chose to analyze this dataset in more detail. It was easy to see that representatives of the male line of the Calments, including Jeanne's father Nicolas Calment, his father, grandfather, and great-grandfather, who were all ship carpenters by profession, and their wives lived long lives. The parents of their wives also lived long. Going further back, it turned out that Vincent Calment's father was a hostler and died at the age of 68 years, and his grandfather was a worker and died at around 50 years. Other ancestors of Vincent also could not boast exceptional longevity.^63^

**FIG. 2.**
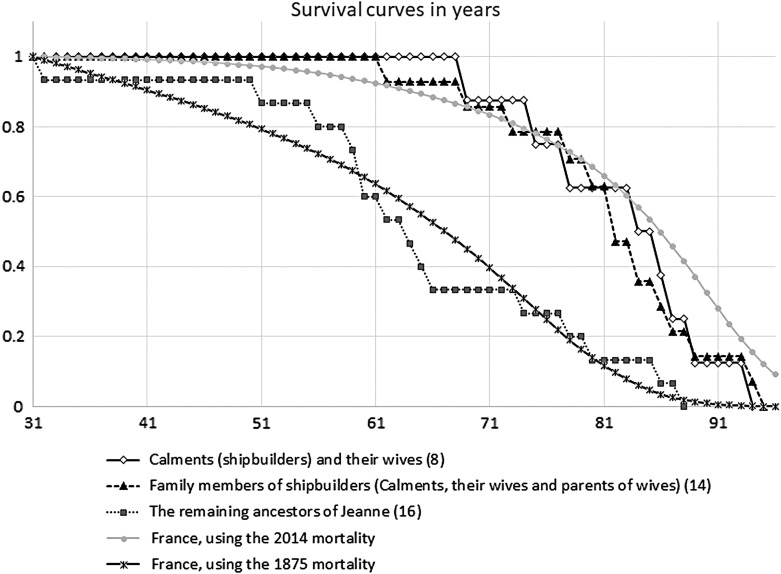
Survival curves of three groups of ancestors of Jeanne Calment. The shipbuilders and members of their families lived longer than the other ancestors just as the French live longer nowadays than in XIX century.

I thus considered three groups of Jeanne's ancestors—the Calments and their wives; the Calments, their wives, and their parents; and Jeanne's other ancestors—and drew the survival curves of these groups, as well as synthetic survival curves based on the death rates in France in 1875 and 2014 from the age of 31, when the first death was recorded (Fig. 2).

It will be seen that the survival curves of the first two groups are almost indistinguishable, and the third group experiences much higher mortality at young ages. Apparently, the longer life span of the family members of ship carpenters was due to a more favorable environment: prosperity, quality of food, the ability to escape from an epidemic by retreating to a country house (during the cholera outbreak in 1884, the Calment family was living in their “mas” in Saint Martin de Crau^21^), and high social standing. The low infant mortality among the Calments' relatives could also have been aided by the presence of a large number of midwives in this family.^14^ But whatever the reason, the above-average longevity of the family seems to arise mostly, if not entirely, from the absence of early-life mortality.

The same conclusion arises if we focus on the longest-lived individuals: none of Jeanne's ancestors reached 95 years.

Finally, analyses of the life spans of twins who were separated and grew up in different families,^18^ of numerous genealogies,^19^ and of complete genomes of supercentenarians^20^ have brought into question the contribution of genetic differences to human life span.

### Publicity (lack of)

Madame Calment managed to stay in the shadows until she moved to a nursing home in 1985 when she was almost 110 years old. Jean-Claude Lamy studied the archive of the local newspaper, Le Provençal, in Arles and did not find any mention of the new centenarian.^21^ Instead, the newspapers wrote about another long-lived person who celebrated her 95th birthday in 1975.^21^ This might have been because local journalists didn't care since they suspected that she wasn't really 100, and/or because Jeanne wanted to minimize media attention so as to protect a secret. The latter explanation is reinforced by the fact that, according to an interview of the former mayor of Arles, Jacques Perrot: when he learned that a woman from Arles was 100 years old, he was going to visit her, invite her family, and bring a gift but received a polite but firm rejection. Madame Calment did not want the ceremony, so the birthday passed unnoticed.^21^ Notably, Jeanne's attitude completely changed in the nursing home, where she adored the press, gifts, and parties and was not confused by the attention of journalists. “I've waited 110 years to be famous. I intend to make the most of it for as long as possible.”^22^ These points become highly relevant when we consider, in the section “The Identity Switch Hypothesis,” just what kind of secret Jeanne might have wanted to protect.

In any event, Jeanne Calment does not satisfy the first criterion of validating long-lived people proposed by Sir William Thoms in his paradigm-shifting book concerning the validation of centenarians^23^ and acknowledged by Robine and Allard^33^: a public declaration of reaching the age of 100 years or recognition of the centenarian in the local press. The validators decided to make an exception for Calment because of the census in 1975, which listed her as born in 1875.

The evidence and analysis presented thus far highlight just how extraordinary it is that Jeanne Calment could have reached her documented age at death. This is important because the degree of implausibility of a hypothesis determines the level of plausibility to which alternative hypotheses must rise to merit objective examination. In the remainder of this article I discuss one such hypothesis.

### The Identity Switch Hypothesis

### Summary of the hypothesis

The alternative scenario that I will scrutinize below is as follows:

- Jeanne Calment actually died in 1934.
- Her family registered the death of Jeanne as the death of her daughter, Yvonne Calment, for financial reasons.
- Yvonne then masqueraded as Jeanne for the rest of her life.
- This switch was known only to a rather small number of people.
- The person ostensibly most likely to have known about the switch and to have had a motive to report it may actually, on closer inspection, not have.

The essence of this scenario (although not the motive; see section “Possible motives”) has previously been described by various researchers,^10,25^ but has always been rejected as too implausible (see section “The Challenge: How Could the Secret Persist for So Long?”). In this study I argue that, especially in view of the considerations noted in the section “Evaluation of the Plausibility of Jeanne Calment's Life Span,” this rejection may have been overly hasty.

### Possible motives

A possible financial motive for the identity switch could be tax evasion. As far as I know, the first time this version of events was expressed was in 2016, in the discussion of Calment's article (not in the article itself) in the French Wikipedia^26^ by the user hbourj (Henri Bourjade). Thanks to Cyril Depoudent,^62^ I obtained a list of 10 real-estate transactions conducted by Jeanne Calment between 1897 and 1955, as well as transactions made by her family members. These include a large rental deal in Arles (probably of the space of the shop which was closed in 1937) of 375,000 francs in 1938 (which is roughly $375,000 today) and the sale of the farm in Saint Martin de Crau, which was formerly owned by Jeanne's father Nicolas Calment, in 1951 for 5.5 million francs, which is equivalent to around $500,000 today. One of the sellers was Jeanne and another was her grandson Frédéric Billot, who had probably bought the share of his uncle François in 1943 for 500,000 francs (∼350,000 modern dollars). This farm was probably inherited by Jeanne and François from Nicolas Calment after his death in 1931, since the only large gift by Nicolas to his children was made in 1926 and amounted to 72,000 francs (∼130,000 modern dollars). After the death of Nicolas in 1931, both Jeanne and François sold some property for 35,000 francs each, and it was stated that Jeanne had sold the apartment at Roquette, 53—the address of the parents of Jeanne Calment (they both had died there, according to the death certificates obtained by Cyril Depoudent^62^ and Jeanne was counted there before her marriage,^58^ see section “Who was born in the ship carpenter's house, 5 Rue du Roure”).

Tax rates rose sharply in the early decades of the 20th century. “Between 1791 and 1901… the proportional tax rates were fairly small (generally 1%–2% for children and spouses), so there was really very little incentive to cheat. The estate tax was made progressive in 1901. In the 1920s, tax rates were sharply increased for large estates. In 1901, the top marginal rate applying to child heirs was as small as 5%; by the mid 1930s, it was 35%; it is currently 40%. Throughout the 20th century, these high top rates were only applied to small segments of the population and assets.”^27^ Interestingly, the tax laws seem to affect the timing of reported deaths.^28^

Perhaps the Calment family suffered from taxation after the death of Maria Felix (widow of the founder of the store, Jacques Calment) and especially after the death of Jeanne's father Nicolas Calment, the owner of land and real estate in the surrounding villages^14,21^ in 1931. The inheritance tax for the farm in Saint Martin de Crau could amount to hundreds of thousands of dollars in modern money. It is not hard to imagine that the family had neither desire nor ability to pay the tax, especially twice in a row (here, one should recall that Jeanne hated socialists^14,21^). Information about paid taxes must be stored in the relevant archives,^27^ so one can try to check the taxes paid after the death of Nicolas in 1931, given access to them. In addition, Jeanne and her husband had a lot of common property (which they had rented out after the closure of the store), the inheritance of which could also be heavily taxed.

Another motive could be the annuity contract presumably signed before 1934,^38,52,53^ see section “Rumors.” This would motivate the switch, as Yvonne masquerading as Jeanne would still receive payments after the death of Jeanne (or, depending on the contract, of both Jeanne and Fernand; Fernand was 65 in 1934). Pension fraud is a major contributor to extreme age claims.^43^

### Suggestive evidence

We now examine a long list of items that are (to varying degrees) surprising in the context of Jeanne Calment having died in 1997 and Yvonne in 1934 but are consistent with the reverse. No one item constitutes a “smoking gun,” but I claim that in combination they form a compelling case. Some of them were already known to previous analysts, but this is the first time that they have been systematically assembled.

#### Mistaken identities

A remarkably large number of examples exist in which Calment or a member of her family describes someone using wording that is incompatible with the no-switch scenario but fits with the switch scenario. While the errors made by Calment may be ascribed to her age, those made by others cannot.

##### Who owned a store?

According to Robine and Allard, “Madame Calment didn't really become famous until 1988, when the centennial of Vincent van Gogh's stay in Arles was celebrated. Without the link to a famous artist, perhaps she would have remained unknown. In 1994, the second-oldest French person died at the age of 113 years without either having been studied or having received national recognition. But Jeanne Calment quickly passed from local fame in Arles to worldwide fame.”^14^ Her reminiscences concerning the family shop, however, incorporate a curious confusion. In the Canadian film “Vincent and me,”^29^ which made Jeanne the oldest-ever movie star, the 114-year-old woman informs a young girl that she met the artist in her “father's” shop. Newspapers also mentioned this “father's” shop,^30^ as well as the GRG website,^31^ and Robert Young on the 110 Club.^32^ However, Jeanne's father Nicolas, unlike Yvonne's father Fernand, never had any store. There were actually no shopkeepers in Jeanne's ancestry^49^ contrary to what was stated in Robine et al., 1998^13^ but shopkeepers were present in the ancestry of Yvonne.

##### Who was named “Gilles”?

Here is another example of confusion: *Gilles, whose name was that?* “It's my grandmother's name, my grandparents on my father's side.”^14^ After some discussion, she corrected herself and said that it was her mother's side; but in fact “Gilles” was the name of Jeanne's maternal grandfather and was thus both the married name of her maternal grandmother and also the maiden name of her mother. Regardless of whether maiden or married names are more often used in such situations, it is somewhat surprising that she would preferentially mention her grandmother rather than her mother.

##### Who lived with Joseph Billot after the war?

According to the 1954 and 1962 censuses, Madame Calment lived together with her son-in-law colonel Joseph Billot, and Frédéric lived with his wife Renée in a nearby apartment.^33^ Jeanne was an unusual mother-in-law; she got along perfectly with Joseph, with whom they previously co-raised Frédéric.^14^ Moreover, in the 1962 census, the marital status of Joseph Billot was corrected: it shows “M” (for “Married”) and “V” (for “Veuf,” widower) typed over each other.^33^

##### Whom did Marthe Fousson accompany to school?

In their report,^33^ the validators write that Jeanne mentioned the servant Marthe Fousson, and they cite the census of 1911 in which she is counted together with the Calment family. However, the only mention of a servant with the name Marthe that I found in the biographies of Calment was the following: *Did you go to school with your friends, or did you go alone?* “My father or the maid accompanied me…” *What was the maid called?* “Marthe, Marthe Touchon.”^14,34^

In the last years of Calment's life when these validation talks took place, she had difficulty with speaking precisely.^14^ On the basis of almost identical-sounding surnames and the coincidence of the name, and taking into account that there was nobody named Marthe living with Jeanne or nearby when she was a child according to censuses, and the only servant found in the census of 1886 was called Marguerite Minaud (who was a relative of Jeanne born without a father and was never mentioned by Calment or her validators), I conclude that Marthe Touchon is highly likely to be the same maid as Marthe Fousson from the validation report. However, according to the archives of the commune of Fontvieille, she was born on March 8th, 1885 and thus was 10 years younger than Jeanne, so she couldn't accompany Jeanne to school. By contrast, she could well accompany her daughter Yvonne.

##### Whom did Césarie Gachon teach piano?

Although Jeanne did not recall the names of her early school teachers, she remembered her first piano teacher (when she was seven) very well. She identified this teacher as “Cesari Gaston,”^14^ and François Hureau discovered that most probably she was in fact Césarie Gachon (also mentioned in Lamy, 2013^21^). But Gachon was only 14 in 1982 when Jeanne was 7. By contrast, when Yvonne was 7 (in 1905), Gachon was 37, and indeed she was listed as a piano teacher in the 1896 census^59^ and a music teacher in the 1911 census.^60^

##### Who did Frédéric think brought him up?

After Yvonne's official death, her son Frédéric was brought up by his grandmother Jeanne Calment. He called her “Manzane,” meaning “mama Jeanne.” According to Cavalié, 1995,^35^ when Frédéric was 1 year old, his parents went for a vacation to Italy, leaving the child to his grandparents. The child persistently tried to call his grandma “mama,” but they reached a compromise. After Yvonne's death, Jeanne treated Frédéric as her child.^14,21,35^

##### Who knew Frédéric Mistral?

In another interview, Calment was asked: “Did you ever meet Frédéric Mistral?” (Mistral received the 1904 Nobel Prize in Literature.) She replied: “Yes! Yes, he was a friend of my father… um, he was a friend of my husband.”^14^

##### Who was the second “Maria”?

According to the 1931 census, the Calments' house on Gambetta Street in Arles was inhabited by homeowner Fernand Calment (Jeanne's husband) along with his mother Maria, his wife “Maria” (the validators suggest that this was a confusion with his mother and should be read as Jeanne), Yvonne's husband Joseph Billot, Yvonne's son Frédéric Billot, and two maids.^33^ The validators explain the absence of Yvonne by a recopying error.^33^ However, an alternative interpretation is that Jeanne's health was by then in decline and the family was already in the early stages of devising an ID-switch plan, leading them to provide obfuscating information.

##### Who lived through the 1884 cholera outbreak?

In her numerous interviews, Madame Calment discusses a lot of hard-to-check details of her childhood, starting from the age of three, but does not mention the epidemic of cholera that devastated Arles in 1884 when Jeanne was 9 years old.^14^ Soviet validators of centenarians from Azerbaijan have frequently used questions about past cholera epidemics for validation purposes^11^ because they have a good track record of being remembered. It is thus surprising that Jeanne initially omitted it, even though the family retreated to Saint Martin de Crau during that period.^21^

##### Who was born in the ship carpenter's house, 5 Rue du Roure?

Another important event of Jeanne's childhood was the moving of her family from Du Roure 5 to Roquette 53 in 1888 when she was 13, as uncovered recently by François Robin-Champigneul^63^ and confirmed by patent,^57^ mortgage^62^ and census^58^ documents. This change of home was so thoroughly absent from Jeanne's reminiscences that the validators said she had only one address before marriage;^33^ moreover, Jeanne stated explicitly in 1988 that she was born at Roquette 53,^54^ where in fact neither Jeanne nor Yvonne was born.^33^ Later, illustrating similar confusion, she stated that she was born at “Du Roure 53” (one home's street, the other's number).^35^

##### Who was the huntress?

According to the biography of Calment,^35^ just before the start of World War I, when Jeanne was almost 40 years old while her daughter Yvonne was around 15, she said that she was very excited to go hunting with Fernand and not being afraid of anything, she acquired the masculine nickname “Jean Calment” and became a member of the hitherto male-only hunting club. After that, she went on to master skis on the glaciers of Grenoble.^35^

However, in another biography, Jeanne states that she was already a huntress when she was 20 years old: “*Do you remember how were you dressed at the time… when you were 20 years old for example*? Oh, you ask too much of me. You go too far! I led a man's life. I was a huntress, you understand. That will surprise you.”^14^

#### Physical appearance

This work was inspired by my conversations with Dr. Valery Novoselov, who argued that Madame Calment was highly suspicious from a point of view of clinical geriatrician. Novoselov is going to discuss her clinical phenotype in a separate study.^36^ Thus, I only briefly summarize that aspect of the evidence here.

**FIG. 3.**
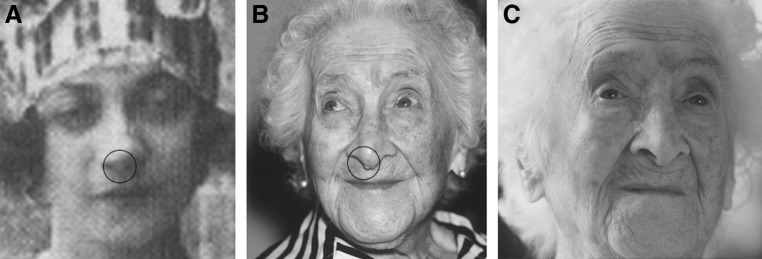
**(A)** Rare photo of young Yvonne Calment scanned from Simonoff, 1995.^42^ Visible feature near the nose tip (circled) could be a fibroma. **(B)** Photo of Calment at the age of 114, reprinted with permission by Sipa Press. Visible feature near the nose tip (circled) is probably a fibroma. **(C)** Photo of Calment at the age of 117, reprinted with permission by Sipa Press. The fibroma is no longer present. Was it also removed periodically at earlier ages? See section “Physical appearance.”

In one of the few photographs of the young Yvonne that exist (Fig. 3A), one can see a small fibroma on the nose (it could be a scan defect, but it is also visible on different scans^54,37^). A similar fibroma can be seen in one of the photos of the old Calment (Fig. 3B; fibromas circled). Interestingly, it is absent from later photos (*e.g.*, Fig. 3C), indicating that it was removed. If Yvonne removed it more than once, that could explain its slightly different locations in Figure 3A and B and also the fact that the fibroma appears smaller in the older woman, even though fibromas grow over time.

In addition, there appears to be inconsistency with regard to Jeanne's eye color in youth versus her later years. In the color version of Figure 3B,C we see that they were green and Michel Allard has confirmed it,^61^ but in the 1930s it was stated that they were black (presumably, dark brown).^35^

Finally, the old Jeanne looked almost unbelievably young for her age. While it is to be expected that those who reach exceptional ages will look similar to considerably younger people (because they “stayed younger longer”), the magnitude of that disparity in this case may be viewed as so extreme as to raise suspicion.

#### Rumors

In addition to many biographies that do not question Jeanne's identity, I located a rare book on insurance published in 2007,^38^ where there is a short paragraph in the chapter on insurance fraud devoted to Madame Calment. This paragraph was based on an article^52^ (kindly sent to me by Benoit Martin) by Jacques de Baudus, who wrote that during the dinner a very respectable guest told nine respectable persons that a state insurance company (identified as Caisse Nationale de Prévoyance [CNP]^53^), which paid a lifetime annuity to Jeanne Calment for a long time, discovered that it was a daughter of Jeanne Calment who received the rent, but, by agreement with the authorities, it kept the matter secret given how much the character of the “doyenne of the French” had by then become legendary. The details of the story are not clear. According to it, Jeanne Calment signed this contract before her death in 1934 (thus creating a potential motive for the ID switch, see section “Possible motives”). Equally, it may be that it was Yvonne who signed the contract later and profited from being younger than her official age (see section “Why was Raffray silent”). An officially sanctioned investigation in the archives of CNP might provide more credibility to this anecdotal evidence.

## The Challenge: How Could the Secret Persist for So Long?

As noted, the ID-switch hypothesis has been mooted by various researchers in the past but has never been given credence. The main basis for rejecting it has probably been the assumption that a large number of people would surely have known about the switch, making it as good as impossible to keep it from the authorities. However, closer inspection reveals that the number of people who knew may have been quite modest.

### Where did Jeanne and Yvonne live during the relevant period?

Jeanne Calment lived in the same house as one of the largest drapery stores in the region, Nouveautés—Calment, which was run by her husband (and cousin) Fernand Calment, so Jeanne should have been constantly seen by the commune. This belief can be traced back to Robine and Allard's validation report on Jeanne Calment's life span,^33^ where they assert that, for the whole time between her wedding and moving to the nursing home, Jeanne had been living in the house where her husband's store was located. Moreover, they write that Nouveautés—Calment still exists at the same location on Gambetta Street. It was hard to imagine how Madame Calment could successfully lie about her age while living in the center of a small town.

However, the commune of Arles is actually the largest commune in France, with a surface almost nine times the surface of Paris. It incorporates several nearby villages where its wealthy inhabitants (including the Calments) used to have country houses. The population of the commune of Arles was around 24,000 people in 1875 and almost doubled by 1997, while there are around 380 inhabitants in a median-sized French commune.

According to her biographies and mortgage documents, Calment had many other places in several communes to live in, and her favorite property was La Miquelette, a villa in the village of Paradou near Arles which Jeanne and Fernand bought in the early 1930s for their daughter Yvonne and her son Frédéric Billot (1926–1963).^35^ There is now only a renewed historical sign on the façade of the Maison Calment, the house where the store was formerly located. Contrary to what is asserted in the official validation,^33^ the drapery store itself has been permanently closed since 1937,^35,39^ and now there is a supermarket, Casino, in that location.

Similarly, the trail of Yvonne Calment went cold after her wedding in 1926. As noted earlier, she was not listed in the 1931 census. She was also not counted with her parents in 1926.^33^ Her husband, Captain Joseph Billot, travelled a lot (in 1926–1928, *e.g.*, according to his military documents^40^ he had tours of duty in Fontainbleau, Toul, Paris, and Bourges). In fact, Calment told her validators that she followed her husband and enjoyed travelling (and hunting) with him.^14^ In the early 1930s, Yvonne may have spent a lot of time outside of Arles, residing in her villas where she could be known under the pseudonym Jeanne. In addition, most people knew her just as Madame Calment, no matter what name she used.

Pierre Maxence is the son of Marius Maxence, the oldest employee of the store Maison Calment, who worked there for about 30 years until its closure in 1937. According to his testimony, Madame Calment almost never appeared there.^39^ The fact that she is mentioned in many censuses at the address of the store does not mean that she lived there all the time. According to Robine and Allard, Jeanne is counted twice in the 1901 census: at St Martin de Crau and in Gambetta Street in Arles.^33^ Similarly, in the 1901 census her brother François was also counted at St Martin de Crau^33^ while also being counted with his wife and daughter in Toulon.^62^ Of note, Robine and Allard write that François had no family and children.^14^ Might this be another case of deliberate obfuscation of census data by the family? Of course it would not be related to the ID switch, since it occurred decades earlier, but it might indicate a pattern of behavior.

### The 1934 funeral

Even if not many people knew Jeanne and Yvonne in the relevant period, there was one event at which it should have been particularly difficult to maintain secrecy: Yvonne's funeral. It was a very public event: Camille Le Pomellec^41^ cites a newspaper report^64^ a week later that an “especially big crowd followed Madame Billot-Calment for her last journey.” However, the cause of death may provide an answer. Yvonne may have suffered from a chronic condition,^35^ specifically tuberculosis, eventually degenerating into pleurisy, for several years before her death^55^—but where? Wealthy people were more often treated at home,^41^ and Le Pomellec failed^41^ to find confirmatory notes at the hospital in Arles, in spite of the following testimony of Madame Calment: “When she came back from hospital, it was Advent and she came to die at home.”^14^ This contradiction would be resolved if she was sent to another location, and indeed rural sanatoria were a popular option for tuberculosis sufferers at that time.^65^ Clearly, that would have helped keep a planned ID-switch secret; indeed, maybe it was Jeanne who had tuberculosis (which would, helpfully, have kept her away from Arles). In line with this, she told her validators “we took her to… Oh! I don't recall any more.”^14^ Death from such a severe infection could also have been an excuse to keep the dead woman's casket closed and to justify the living woman wearing a heavy veil. Camille also notes that unlike her mother's, son's and husband's names, Yvonne's name is not present on the tombstone of the familial grave.^41^

### Timing and social norms

The late 1930s were turbulent times in Europe. World War II brought chaos with it,^21,35^ and after the war it may have been relatively easy to promulgate the fiction that Madame Calment was always Madame Jeanne Calment.

Moreover, one should also take into account the specifics of Provence, which was a region with its own language and mentality where (as everywhere in France) the idea of paying huge taxes to the French Treasury could be not very popular and corruption at the local level may not have been uncommon.

It is also somewhat suggestive that Jeanne decided to destroy the photos and other documents when she was requested to send them to the archives of Arles.^21^ Being in the nursing home and not being able to destroy the documents herself, Jeanne resorted to the help of a distant relative.^42^ (Intriguingly, that relative—a niece of Joseph Billot named Madame Bigonnet—was Calment's heiress, so she had multiple motives to help perpetuate an ID-switch secret.) Robine and Allard address this point but downplay its significance, as follows: “While she was still in the Maison du Lac, Madame Calment destroyed all her personal archives, all her family photographs. That's a bit surprising; yet, this attitude is not so unusual. Many people in her situation, without direct descendants or close friends, decide, in the twilight of their lives, to leave nothing ‘dragging’ behind them–nothing that affects them, no trace of their existence… She would say: ‘I don't want my image scattered.’ ”^14^ However, in the context of the pattern of behavior documented here, it becomes plausible that this destruction was a result of cold calculation and acute necessity instead of an emotional act.

Finally, there is evidence that the family was not socially active after the war. Claudine Serena, who nursed Madame Calment during her last years, told Camille Le Pomellec that Madame Calment despised ordinary people and people disliked her.^41^ Such a character would help her under the ID-switch scenario by minimizing contact with those who might not be trusted to keep the secret if they discovered it. When one journalist asked Madame Calment: “You were a charming woman; you must have had a lot of gallant proposals?” she answered him: “My husband was a marvelous man. When one has an angel, one keeps him, and the rest don't see you.”^14^

### Why was Raffray silent?

Under the ID-switch hypothesis, a manageably small inner circle knew about the substitution but did not tell anyone. The circle consisted of the family themselves, who obviously had a financial interest in perpetuating the deception, and also a number of people who were close friends of the family and thus would be expected to have kept the secret merely out of loyalty. For example, a 71-year-old widow, Joséphine Audibert (born in Tarascon), claimed to have seen Yvonne's corpse, and her testimony was signed by the aide of the mayor of Arles, Justin Valle, who claimed to have ascertained the fact that Yvonne Calment died at 2:00 a.m. on January 19, 1934, at the age of exactly 36 years at her home on Gambetta Street.^62^ Doubtless a number of other friends and colleagues knew the truth but viewed it as not their business (and, perhaps, as socially inexpedient) to whistle-blow on a family with substantial wealth and influence in the region. But there was one individual for whom these arguments may initially appear insufficient.

One of the most famous stories about Jeanne is that she settled a life annuity deal with the notary André-François Raffray,^51^ and he apparently had no doubts about her age.^34^

Raffray agreed to pay 2500 francs monthly in exchange for acquiring her apartment in the center of Arles, where she had been counted in censuses since her marriage in 1896. The annuity was calculated according to the life expectancy of 90-year-old French women in 1965, which was around 3 years. (There is some confusion concerning who owned the annuity at what time; it seems to have been owned by the widow of Jeanne's grandson between 1967 and 1969 when Raffray acquired it from her^51^ but many other sources state that it was first signed when Jeanne was 90, i.e in 1965).^34^ Thus, the notary (and, after his death in 1995, his widow Huguette Raffray) had to pay 10 times more than expected.^51^ Huguette informed me by phone that the apartment was sold immediately after the death of Jeanne, and she does not know who the buyer was.

This initially seems to be a big challenge to the ID-switch hypothesis. Since Raffray moved to Arles only in 1959,^56^ perhaps he didn't know (but see the different explanation below) about the switch at the time he entered into the agreement (since then the expected time until he received the property would be many times longer). However, we cannot ignore the possibility that he discovered it later. At that point, he could presumably have terminated his liability in an instant by revealing the switch to the authorities. Were the social pressures noted above so strong as to be worth losing so much money? It seems unlikely.

But two explanations are easy to posit that are fully compatible with the ID-switch scenario. Firstly, at the time of Raffray's (putative) discovery of the switch, Yvonne's reliance on perpetuating the secret would have been enormous: probably the threat of a large tax bill would have reached a statute of limitations, but there might have been the prospect of a criminal trial, or at very least a huge embarrassment for Yvonne personally. Moreover, as noted in the Rumors section Jeanne received a large rent from the government company; this would certainly have been revisited if the company had been told about the switch at that time (since Jeanne was not yet famous then).^51,53^ Thus, she had both a motive and the ability to repay Raffray “under the table” in order to buy his silence.

Alternatively, it is equally reasonable that Raffray was indeed aware of the switch before signing the deal, but was friendly with Yvonne, so they signed the deal (along with the understanding that his payments would be covertly returned) precisely to make the ID switch seem less plausible to the wider world, and less likely to be investigated by those who would have suffered financially from it.

## Conclusion: Lessons for the Future

Forensic comparison of the bodies and DNA of family members buried in the familial grave could potentially be performed to check whether there was an identity switch. In fact, it is also possible that biological material from the person who died in 1997 is still in storage: cells belonging to her were probably taken for analysis by the project Chronos^42^ and maybe some other projects (there was even some information on her HLA group in Wikipedia). Curiously, Jeanne and Fernand were double second cousins while there was no consanguinity detected in at least four generations of Jeanne's ancestors^14,33,34^ and thus Yvonne is expected to have significantly higher level of homozygosity^50^ than Jeanne if their official genealogy^49,34^ is correct. Thus one can test the validation of Jeanne Calment's age by analyzing the DNA from cells taken from Madame Calment late in life. It is unfortunate that Calment's medical card is not available for study^21^ and her last geriatrician, Dr. Catherine Levraud, refused to provide me with any medical information; political will may be needed to clarify these matters.

In addition, there is a case for expert analysis of surviving photos of Jeanne and Yvonne to arrive at a firm determination of who is pictured, since various commentators have suggested that the old Jeanne looks more like the young Yvonne than the young Jeanne and found multiple confusions in naming of photos of Yvonne and Jeanne in newspaper articles published while Jeanne was a recognized supercentenarian.

More broadly, however, we must ask what can be learned from the fact that more than 20 years passed with (as I have argued here) severely inadequate scrutiny of what is probably the single most famous data point in the entire field of gerontology. According to Norris McWhirter, the editor of the Guinness Book of World Records, “No single subject is more obscured by vanity, deceit, falsehood and deliberate fraud than the extremes of human longevity.”^43^

It should first be noted that the science of extreme longevity is becoming more rigorous. In the past, oral testimony was enough to recognize the age of a person, but now multiple pieces of documentary evidence are required for validation, and numerous myths have been debunked.^23,24,43,44^ Robert Young, the director of the GRG, a consultant for the Guinness Book of Records and the administrator of the 110 Club, writes that in cases when the declared age is approaching 120 years, the validation should be very thorough, as was done in the case of Jeanne Calment. To stress this point, a list of false examples of long-lived people is provided in Young et al., 2010.^43^ But there is still room for improvement, as illustrated not only by the Calment case but also by the following examples.

One of the well-known examples of disproved supercentenarians, Pierre Joubert,^24^ who died in 1814 at the age of 113, was not questioned until the end of the 20th century, when the demographer Hubert Charbonneau showed that Pierre Joubert had in fact died in 1766 and it was his son, born in 1732, who has been mistakenly considered as the oldest Canadian for a long time. It is important to note that in this and other false examples of long-lived people,^43,44^ the substitution was not pre-planned, so it was quite easy to expose (Charbonneau found the real death record of Pierre Joubert). In the case of Jeanne Calment, on the other hand, we are talking about a well-planned identity switch in the distant past of 1934.

For obvious reasons, among claimed supercentenarians, there should be a disproportionately large number of people whose documents contain intentional or accidental errors that overstate their age. On February 21st, 1986, when Jeanne Calment turned 111 years old, the previously universally recognized record holder, Shigechiyo Izumi, died at the claimed age of 120. It was eventually discovered, however, that Izumi acquired the birth certificate of his deceased older brother, and, in fact, he lived “only” until 105.

Two other universally recognized rivals of Jeanne Calment were also recently “devalidated”: 117-year-olds Lucy Hannah, for whom researchers at the 110 Club showed that the census data referred to another person, and Carrie White (it turned out that a mistake by an employee of a psychiatric hospital led to an overstatement of the age of Carrie by 14 years).

The significance of such errors is disproportionate to their number. Jean-Marie Robine, one of the validators of Jeanne Calment, and his colleagues from the IDL note that “Studying extreme ages necessarily involves small numbers, and therefore, a single age error, especially at the highest ages, may well have strong implications for predicting the trajectory of mortality. It is thus of great importance to check the reliability and accuracy of each reported age from such a dataset.”^9^

There are also quantitative ways to see that errors in a small fraction of birth and death records can significantly distort the observed dynamics of mortality. Even if errors are distributed normally, survivor bias means that the observed force of mortality can plateau or even decrease in old age, even if in reality, it is increasing exponentially.^45,46^ This is in line with ideas expressed by Brouard.^47^

However, the mortality plateau in humans may still exist. To ascertain this more rigorously, it is necessary to revisit validation principles and try to create a small, representative (the probability of being recorded should not depend on age—this principle was not taken into account in Gavrilova et al., 2017^3^ as was explained in Zak, 2018),^7^ and truly verified list of semisupercentenarians (individuals over 105 years old). If, in addition to participating in validation, geriatricians become involved in the study of these long-lived people and collect longitudinal data on their health, the analysis of such data will help in understanding the process of individual aging. It is also necessary to avoid any conflict of interest. Selective revalidation of existing supercentenarians will allow estimation of the frequency of fake cases. It would be highly valuable to develop and implement objective methods of age estimation with narrow confidence intervals using molecules with negligible turnover rate in the human body (see e.g. Lynnerup et al. 2008).^48^

In summary: the cumulative weight of evidence presented in this study leads me to the conclusion that Jeanne Calment probably never became a centenarian, let alone a “double supercentenarian.” I conclude that the scientific community should cease to consider her as the validated oldest human until additional tests are done to reject the identity-switch hypothesis.

The name Calment is probably one of the most frequently mentioned in books and articles on the topic of aging. The study of this story can be useful not only for gerontologists but also for scientists in general, as it shows the potential fragility of common knowledge.
